# Hormonal therapy might be a better choice as maintenance treatment than capecitabine after response to first-line capecitabine-based combination chemotherapy for patients with hormone receptor-positive and HER2-negative, metastatic breast cancer

**DOI:** 10.1186/s40880-016-0101-7

**Published:** 2016-04-25

**Authors:** Xue-Lian Chen, Feng Du, Ruo-Xi Hong, Jia-Yu Wang, Yang Luo, Qing Li, Ying Fan, Bing-He Xu

**Affiliations:** Department of Medical Oncology, Cancer Hospital, Chinese Academy of Medical Sciences & Peking Union Medical College, Panjiayuan, Chaoyang District, Beijing, 100021 P. R. China

**Keywords:** Hormonal therapy, Maintenance capecitabine monotherapy, First-line capecitabine-based combination chemotherapy, Metastatic breast cancer

## Abstract

**Background:**

Both hormonal therapy (HT) and maintenance capecitabine monotherapy (MCT) have been shown to extend time to progression (TTP) in patients with metastatic breast cancer (MBC) after failure of taxanes and anthracycline-containing regimens. However, no clinical trials have directly compared the efficacy of MCT and HT after response to first-line capecitabine-based combination chemotherapy (FCCT) in patients with hormone receptor (HR)-positive and human epidermal growth factor receptor 2 (HER2)-negative breast cancer.

**Methods:**

We retrospectively analyzed the charts of 138 HR-positive and HER2-negative MBC patients who were in non-progression status after FCCT and who were treated between 2003 and 2012 at the Cancer Institute and Hospital, Chinese Academy of Medical Sciences, in Beijing, China. The median number of first-line chemotherapy cycles was 6 (range, 4–8); combined agents included taxanes, vinorelbine, or gemcitabine. Of these 138 patients, 79 received MCT, and 59 received HT. Single-agent capecitabine was administered at a dose of 1250 mg/m^2^ twice daily for 14 days, followed by a 7-day rest period, repeated every 3 weeks. Of the 59 patients who received HT, 37 received aromatase inhibitors (AIs), 8 received selective estrogen receptor modulators (SERMs), and 14 received goserelin plus either AIs or SERMs. We then compared the MCT group and HT group in terms of treatment efficacy.

**Results:**

With a median follow-up of 43 months, patients in the HT group had a much longer TTP than patients in the MCT group (13 vs. 8 months, *P* = 0.011). When TTP was adjusted for age, menopausal status, Karnofsky performance status score, disease-free survival, site of metastasis, number of metastatic sites, and response status after FCCT, extended TTP was still observed for patients in the HT group (hazard ratio: 0.63; 95% confidence interval: 0.44–0.93; *P* = 0.020). We also observed a trend of overall survival advantage for patients in the HT group vs. patients in the MCT group, but the difference was not significant (43 vs. 37 months, *P* = 0.400). In addition, patients in the HT group generally tolerated the treatment well, whereas patients in the MCT group experienced grades 3–4 adverse events, the most frequent of which were hand-foot syndrome (15.8%) and hematologic abnormalities (7.6%).

**Conclusion:**

For HR-positive and HER2-negative MBC patients, HT might be considered a treatment after response to FCCT but prior to MCT as a long-term administration.

## Background

The primary goals of metastatic breast cancer (MBC) treatment are to palliate symptoms, preserve quality of life, delay tumor progression, and extend overall survival (OS), not to cure the disease. For hormone receptor (HR)-positive and human epidermal growth factor receptor 2 (HER2)-negative patients with MBC, chemotherapy is needed as first-line treatment when hormonal therapy (HT) has been exhausted or symptomatic visceral metastasis is observed. When the disease is controlled by chemotherapy, several sequential strategies can be pursued: discontinuing chemotherapy and observing until progression; continuing the same therapy; maintaining with a single, mild agent that was used in the initial therapy; or switching to other drugs that are presumed to be effective. Several trials showed that maintenance combination chemotherapy extended the duration of remission [1–7], but modest OS benefits were seen in only two trials [3, 4], and the optimal duration of treatments was not determined. In addition, in most studies, incidence of toxicity was significantly increased. In a Korean phase III trial (KCSG-BRO7-02), patients in the paclitaxel/gemcitabine maintenance arm had much longer median progression-free survival (PFS) and OS than patients in the observation arm (PFS: 7.5 vs. 3.8 months, *P* = 0.026; OS: 32.3 vs. 23.5 months, *P* = 0.047) [4]. The results, however, were debatable because any treatment regarding to HR status was not allowed in the control group after completing six cycles of induced chemotherapy. At the same time, the rate of grade 3 or higher neutropenia for patients in the maintenance arm was as high as 61%, which inevitably resulted in more hospital visits [4]. In fact, many clinicians prefer to discontinue chemotherapy after 6–8 cycles when the disease enters non-progression status. However, it seems inappropriate to just wait for tumor progression. Switching to a more tolerable chemotherapy, such as monotherapy or antiangiogenic agents, might be a better treatment strategy.

Studies have suggested that maintenance monotherapy could be a low-toxicity intervention that significantly extends time to progression (TTP) and has potential OS benefits [8–11]. Since, for many years, anthracyclines or taxanes have been a mainstay of adjuvant therapy for breast cancer, the National Comprehensive Cancer Network has recommended capecitabine-based chemotherapies, such as capecitabine plus taxanes and capecitabine plus vinorelbine, as first-line chemotherapy for MBC patients after failure of taxanes and anthracycline-containing regimens and for patients who cannot receive further anthracycline therapy. In prospective, randomized phase II/III clinical trials, capecitabine monotherapy has shown substantial antitumor activity in the first-line treatment of patients with MBC. According to reports in the literature, patients who received first-line capecitabine monotherapy had a median TTP of 6.0–7.9 months [12]. Long-term administration of capecitabine is convenient and relatively economical and does not result in cumulative toxicity. It still works well in combination with target therapy [12–17]. Therefore, capecitabine is assumed to be a good option for maintenance monotherapy after first-line capecitabine-based combination chemotherapy (FCCT).

For patients with HR-positive and HER2-negative MBC, HT is also an attractive alternative, at least in patients with potentially endocrine-responsive diseases that have entered non-progression status after first-line chemotherapy. In several reported studies of HT with powerful endocrine agents [aromatase inhibitors (AIs) or tamoxifen], the median TTP of patients was 14.4–18.5 months after previous chemotherapy (first- or second-line) [18, 19]. There seemed to be a TTP disparity between the maintenance capecitabine monotherapy (MCT) and HT groups, but we must be cautious that the patient populations of the various studies might be completely different. Previous studies of HT enrolled patients with HR-positive MBC exclusively, whereas studies of MCT involved the whole population regardless of hormone status. Unquestionably, HR-positive patients have a better prognosis than HR-negative patients. Moreover, for patients who are recommended first-line chemotherapy, disease progression is more aggressive than for those who adopt first-line HT. This indicates that the population who are suggested FCCT may be more likely to benefit from chemotherapy. Until now, because no head-to-head comparisons or retrospective studies have been performed between maintenance chemotherapy and HT after disease control, no systematic assessment data exist on the efficacy of maintenance therapy. Therefore, in the present study, we examined HR-positive/HER2-negative patients with MBC after response to FCCT, divided them into HT and MCT treatment groups, compared the efficacy of distinct treatments, and sought to determine which treatment was superior.

## Patients and methods

### Patient selection

We reviewed the charts of patients diagnosed with MBC between 2003 and 2012 at the Cancer Hospital & Institute, Peking Union Medical College, Chinese Academy of Medical Sciences, China. Eligibility criteria for this study were as follows. (1) Breast cancer patients were confirmed as having a primary HR-positive and HER2-negative tumor. Estrogen receptor or progesterone receptor was considered “positive” when at least 1% of the nuclei was stained as determined by immunohistochemical analysis. HER2-negative tumors were scored as 0 or +1 by immunohistochemical analysis or scored +2 but the result of fluorescence in situ hybridization was negative. (2) Breast cancer recurred with measurable metastatic disease. Moreover, response to FCCT was evaluated as complete response (CR), partial response (PR), stable disease (SD), or progressive disease (PD) according to the Response Evaluation Criteria in Solid Tumors (RECIST) criteria, version 1.0. (3) Combined agents in FCCT included taxanes, vinorelbine, or gemcitabine and were administered for at least 4 but no more than 8 cycles. (4) The duration of non-progression status was at least 4 weeks for patients in the MCT group after the last cycle of FCCT, or else it was considered a failure of combination chemotherapy or resistance to capecitabine. (5) Patients’ Karnofsky performance status (KPS) scores were ≥70. Key exclusion criteria were (1) the presence of brain metastases, (2) the presence of immeasurable disease, and (3) the administration of HT in the metastatic setting before FCCT. Informed consent was obtained from all eligible patients. The follow-up and data collection were completed on March 31, 2015.

### Treatment and evaluation

Capecitabine was administrated at a dose of 1250 mg/m^2^ twice daily on days 1–14 at initiation, followed by a 7-day rest period, repeated every three weeks. Dose modifications were made according to the drug instructions or the attending physician’s judgement. Maintenance HT included tamoxifen, AIs, or the combination of ovarian suppression with AIs or tamoxifen. TTP was defined as the interval from the date of administration of maintenance therapy to tumor progression. OS was defined as the time between the initiation of maintenance therapy to death from any cause, with censoring of the last visit date. Additionally, toxicity profiles were assessed and graded according to the Common Terminology Criteria for Adverse Events, version 3.0. Tumor evaluation was performed every 2–3 months according to RECIST 1.0.

### Statistical analysis

Clinicopathologic characteristics were compared between the two groups by the Chi square test. TTP and OS were estimated using the Kaplan–Meier method and compared using the log-rank test. Hazard ratios [two-sided 95% confidence interval (CI)] were calculated with unadjusted and adjusted Cox proportional hazards models for group comparisons. Potential clinical covariates were used to investigate the association of the prognostic factors with TTP and OS. In this model, the covariates included age (≤50 or >50 years), menopausal status (premenopausal or postmenopausal), KPS score, duration of disease-free survival (DFS) (≤2 or >2 years), site of metastases (viscera or non-viscera), number of metastases (1 or >1), and response after FCCT (CR + PR or SD). *P* values less than 0.05 were considered statistically significant. All statistical analyses were performed using SPSS 22.0 software (IBM SPSS, Armonk, NY, USA).

## Results

### Patient characteristics

A total of 257 HR-positive and HER2-negative MBC patients received FCCT, and 174 patients among them were under diseases control. Among the 174 patients, 14 received palliative HT concurrent with FCCT, 16 received maintenance combination chemotherapy, 5 underwent paclitaxel maintenance therapy, and 1 received gemcitabine maintenance therapy. Thus, only 138 patients were eligible for further analysis, with 79 in the MCT group and 59 in the maintenance HT group. At the initiation of maintenance therapy, the median age of the 138 eligible patients was 50 years. Almost all the patients (133 of 138) received a taxane or anthracycline-containing regimen as neoadjuvant or adjuvant therapy, and 122 patients received adjuvant endocrine therapy. Median exposure to initial FCCT was six cycles (range, 4–8 cycles). In the HT group, 37 patients received AIs, 8 received selective estrogen receptor modulators (SERMs), and 14 received goserelin plus either AIs or SERMs. The baseline characteristics of the patients were balanced (except for an imbalance in the number of metastatic sites and response status after FCCT) between the two groups (Table 1). The percentage of patients with multi-site metastases (72.1% and 49.1%, *P* = 0.006) or a better response to FCCT (59.1% and 47.4%, *P* = 0.009) was higher in the MCT group than the HT group.

**Table 1:** Demographic and baseline characteristics of 138 patients with metastatic breast cancer

Characteristic	HT group (*n* = 59)	MCT group (*n* = 79)	*P* value
Age (years)^a^			0.009 (*t* test)
Median	49	50	
Range	37–76	34–66	
KPS score			0.349
90–100	46 (78.0)	56 (70.9)	
70–80	13 (22.0)	23 (29.1)	
Menopausal status			0.520
Premenopausal	20 (33.9)	31 (39.2)	
Postmenopausal	39 (66.1)	48 (60.8)	
HR status			
ER-positive	55 (93.2)	73 (92.4)	0.855
PgR-positive	51 (86.4)	65 (82.2)	0.509
Adjuvant HT			0.246
Yes	50 (84.7)	72 (91.1)	
No	9 (15.3)	7 (8.9)	
DFS (years)			0.446
<2	16 (27.1)	17 (21.5)	
≥2	43 (72.9)	62 (78.5)	
Median^a^ (months)	44	42	0.178 (*t* test)
No. of metastases			0.006
<2	30 (50.9)	22 (27.9)	
≥2	29 (49.1)	57 (72.1)	
Metastatic sites			0.168
Viscera	32 (54.2)	52 (65.8)	
Non-viscera	27 (45.8)	27 (34.2)	
Prior adjuvant CT			
Anthracycline	54 (91.5)	70 (88.6)	0.749
Taxanes	32 (54.2)	53 (67.1)	0.170
Response to FCCT			0.009
CR + PR	28 (47.4)	55 (69.6)	
SD	31 (52.3)	24 (30.4)	

*HT* hormonal therapy, *MCT* maintenance capecitabine monotherapy, *KPS* Karnofsky performance status, *HR* hormone receptor, *ER* estrogen receptor, *PgR* progesterone receptor, *DFS* disease-free survival, *CT* chemotherapy, *FCCT* first-line capecitabine-based combination chemotherapy, *CR* complete response, *PR* partial response, *SD* stable disease

^a^Except for these values, others are presented as the numbers of patients followed by the percentages in the parentheses

### Efficacy assessment of maintenance therapy

**Fig. 1:**
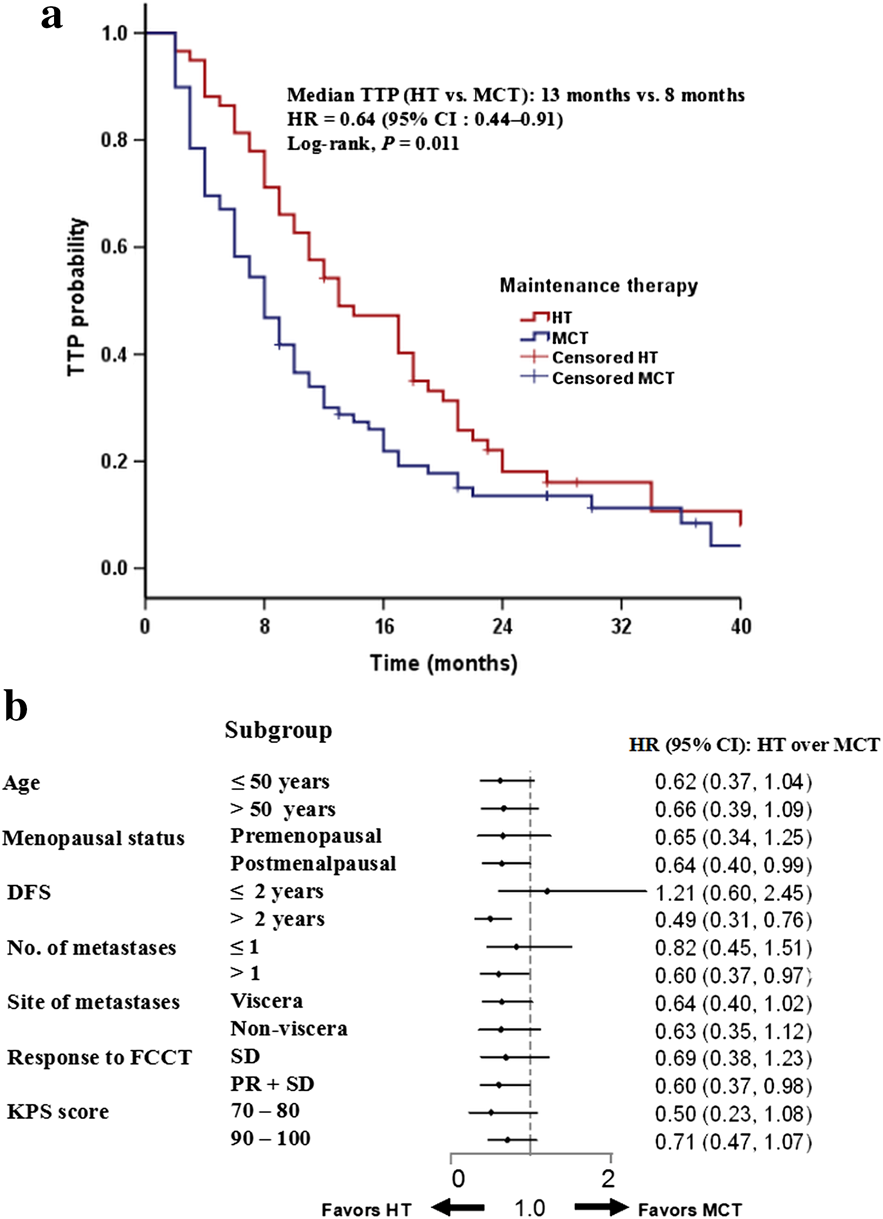
Comparison of time to progression (TTP) between the HT group and the MCT group. **a** Estimation of TTP, censored on the date of last assessment. **b** *Forest plot* (TTP analysis); HR < 1 favors HT. *DFS* disease-free survival, *FCCT* first-line capecitabine-based combination chemotherapy, *KPS* Karnofsky performance status, *CR* complete response, *PR* partial response, *SD* stable disease, *HT* hormonal therapy, *HR* hazard ratio, *CI* confidence interval, *MCT* maintenance capecitabine monotherapy

With a median 43-month follow-up, 51 of 59 patients in the HT group and 72 of 79 patients in the MCT group experienced PD. By the cutoff date, the median TTP in the HT group was significantly longer than that in the MCT group (13 vs. 8 months, *P* = 0.011; Fig. 1a). Although multi-site metastases or a better response (CR + PR) to FCCT were shown in a higher proportion of patients in the MCT group compared with those in the HT group, they were not associated with TTP in univariate analyses (Table 2). A secondary analysis adjusting TTP to predefined covariates (including number of metastases and response to FCCT) still showed improvement in the HT group (hazard ratio: 0.63; 95% CI, 0.44–0.93; *P* = 0.020; Table 3).

**Table 2:** Log-rank analysis of TTP and OS in the 138 patients who received maintenance therapy

Variable	Median TTP (months)	*P* value	Median OS (months)	*P* value
Age (years)		0.516		0.609
≤50	10		42	
>50	9		36	
Menopausal status		0.306		0.076
Premenopausal	10		45	
Postmenopausal	10		37	
KPS score		0.916		0.039^a^
70–80	8		33	
90–100	8		44	
DFS (years)		0.670		0.077
≤2	10		34	
>2	8		43	
No. of metastases		0.081		0.114
≤1	14		55	
>1	9		36	
Site of metastases				
Viscera	9	0.106	41	0.905
Non-viscera	13		42	
Response to FCCT		0.883		0.337
SD	10		41	
CR + PR	10		43	

*TTP* time-to-progression, *OS* overall survival, *KPS* Karnofsky performance status, *CR* complete response, *PR* partial response, *SD* stable disease, *DFS* disease-free survival, *CT* chemotherapy, *FCCT* first-line capecitabine-based combination chemotherapy

^a^Since there were only 36 patients in the group whose KPS score was 70-80, no conclusions could be drawn from the log-rank analysis of OS

**Table 3:** Efficacy analysis of time to progression and overall survival by unadjusted and adjusted Cox proportional hazards models

Variable	TTP	OS
	HT vs. MCT	HT vs. MCT
Median (months)	13 vs. 8	43 vs. 37
HR by unadjusted Cox regression	0.64	0.82
95% CI	0.44–0.91	0.50–1.32
Log-rank *P* value	0.011	0.400
HR by adjusted^a^ Cox regression	0.65	0.83
95% CI	0.43–0.93	0.49–1.37
*P* value	0.018	0.450

*HR* hazard ratio, *CI* confidence interval, *HT* hormonal therapy, *MCT* maintenance capecitabine monotherapy

^a^Adjusted to menopausal status, Karnofsky performance status, number of metastasis sites, interval of disease-free survival, visceral disease, and response to FCCT, which were all predefined in the study protocol

Subset analyses of TTP corroborated the primary overall analysis. The advantage of HT over MCT was maintained across various subgroups. A hazard ratio <1 score favored the option of HT (Fig. 1b). Patients in postmenopausal status (hazard ratio: 0.64, *P* = 0.036), with an interval of DFS longer than 2 years (hazard ratio: 0.49, *P* = 0.001), and with two or more metastases (hazard ratio: 0.60, *P* = 0.037) could get benefits from HT. Importantly, for patients who got a better response (CR or PR) to FCCT, median TTP was still longer in the HT group than in the MCT group (hazard ratio: 0.60, *P* = 0.033).

**Fig. 2:**
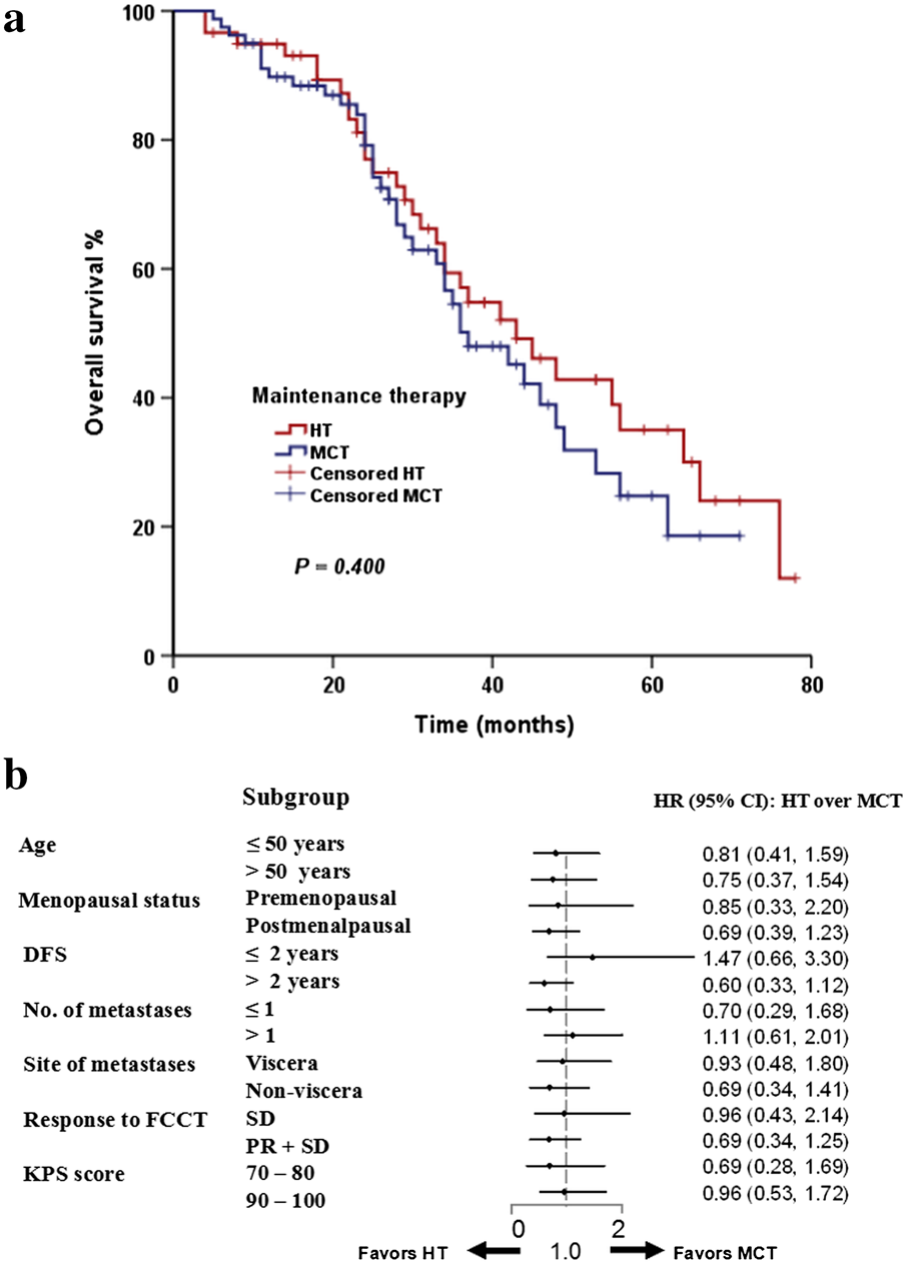
Comparison of overall survival between the HT group and the MCT group. **a** Estimation of overall survival, censored on the date of last assessment. **b** *Forest plot* (OS analysis); HR < 1 favors HT. *DFS* disease-free survival, *FCCT* first-line capecitabine-based combination chemotherapy, *KPS* Karnofsky performance status, *CR* complete response, *PR* partial response, *SD* stable disease, *HT* hormonal therapy, *HR* hazard ratio, *CI* confidence interval, *MCT* maintenance capecitabine monotherapy

A total of 70 breast cancer-related deaths were observed in our study. The median OS of the whole population was 41 months. Similar to TTP, we compared OS between the two groups. We observed a trend of gained advantage in OS in the HT group compared with the MCT group, but the difference in OS was not statistically significant between the two groups (43 vs. 37 months, *P* = 0.400; Fig. 2). The same conclusion could be drawn as OS was adjusted to predefined potential prognostic factors in multivariate analysis. Longer DFS (>2 years) was indicated as a sole independent prognostic factor of OS (hazard ratio: 1.78, *P* = 0.027; data not shown). Exploratory analyses on OS were conducted across predefined subsets. No significant difference was observed indicating specific options of maintenance therapy.

### Safety and tolerance

In this study, we collected data on severe adverse events. During initial FCCT treatment, the most frequently recorded grades 3–4 adverse events were hematologic abnormality (31.8%), hand-foot syndrome (8.0%), and gastrointestinal toxicity (5.0%). During the maintenance treatment phase, 15 of 138 patients experienced grade 3 or worse adverse events. In the MCT group, the most frequently recorded grades 3–4 adverse events were hand-foot syndrome (15.8%) and hematologic abnormality (7.6%). HT was generally well tolerated. No grade 3 or higher gastrointestinal adverse effects were recorded. Only one patient changed to another HT due to vaginal bleeding.

### Sequential treatment

By the cutoff date, 7 patients in each group were continuing their maintenance therapy. The remaining 72 patients in the MCT group received second- or third-line HT after disease progression, whereas only 34 (66.7%) patients received capecitabine in their sequential treatments.

## Discussion

In this study, we examined HR-positive and HER2-negative MBC patients after response to FCCT and found that the HT group had a much longer TTP than the MCT group. The efficacy was prominent regardless of age, interval of DFS, menopausal status, KPS score, number of metastases, visceral metastases, and response to first-line chemotherapy. We observed the same trend in OS, too. Moreover, HT also demonstrated a better safety profile. Therefore, we found that HT was a better treatment option for a subgroup of patients in this setting.

Unquestionably, HT plays an important role in the treatment of HR-positive breast cancer. Because HT has been shown to be remarkably efficacious in the adjuvant, neoadjuvant, and first-line palliative treatment of breast cancer, it is widely used in routine clinical practice during breaks in palliative chemotherapy for women with recurrent or metastatic disease characterized by HR-positive tumors [20, 21]. When HT is administered long term, therapeutic gains are expected due to its efficacy and good safety profile. However, we must be cautious in administering it to all HR-positive patients. Evidence-based data are lacking regarding its maintenance after disease control by previous chemotherapy. To date, only two small prospective trials have investigated the efficacy of maintenance HT after previous palliative chemotherapy. In one study, 90 patients with disease control after 6 cycles of anthracycline- and ifosfamide-containing regimens were randomized to receive maintenance medroxyprogesterone acetate (MPA) therapy or placebo. A longer median TTP was seen in the MPA arm compared with that in the placebo arm (4.9 vs. 3.7 months, *P* = 0.02) [22]. However, this study was conducted in 1990s, and currently MPA is rarely used as first-line endocrine therapy. In a letrozole-based single-arm phase II study, the median TTP from the initiation of letrozole after induced chemotherapy was as long as 18.5 months [18].

As with other orally administered drugs, capecitabine has been extensively evaluated in both pretreatment and first-line treatment for breast cancer patients. Adverse events are readily managed by dose modification. When using capecitabine as first-line treatment for MBC, the objective response rate was as high as 30%–36% for anthracycline- and/or taxane-resistant MBC [13–16]. Si et al. [23] reported that patients who received MCT after FCCT had a median TTP as long as 9.4 months, which is superior to the 4.5 months reported in the observation arm. Clinicians may prefer MCT in practice, expecting sustainability of its good response to FCCT regardless of HR status.

In our study, the survival results of each group were similar to those previously reported, which justified the option of HT for patients with HR-positive and HER2-negative MBC after response to FCCT. To our knowledge, ours is the first study to directly compare the efficacy of MCT with that of HT in this subgroup of patients. Our study did, however, have some limitations, mainly that it had a small sample size and was retrospective in nature, so it is not possible to conclusively say that HT contributed to extended TTP. Since most tumors develop resistance to endocrine therapies after two or three endocrine regimens, the effectiveness of these agents in later-line settings is limited. As reported in the literature, in most studies the median TTP of patients who underwent endocrine therapy as second-line or further treatment was 3–6 months [10], similar to that of whom underwent MCT in later lines [24]. Interestingly, in our study we observed a patient who maintained MCT for 41 months without progression after PR to FCCT. Therefore, we speculate that MCT may also be an appropriate option for a small subgroup of patients with HR-positive breast cancer and may be more effective in later settings. However, this warrants additional phase III clinical trials.

Currently, investigators trying to overcome endocrine resistance are studying many targeted agents, some of which have been found to be effective in the treatment of HR-positive MBC, including everolimus, cyclin-dependent kinase 4/6 inhibitors, and histone deacetylase inhibitors [25]. In a relatively short period of time, options for maintenance HT after previous chemotherapy will be more varied and powerful.

## Conclusion

We conclude that maintenance HT may be considered prior to administration of capecitabine after response to FCCT for patients with HR-positive and HER2-negative MBC.
